# Strategies for Considering Environmental Justice in the Early-Stage Development of Circular Economy Technologies

**DOI:** 10.1021/acssuschemeng.4c02205

**Published:** 2024-05-21

**Authors:** Taylor Uekert, Julien Walzberg, Hope M. Wikoff, Meredith M. Doyle, Alberta C. Carpenter

**Affiliations:** † Strategic Energy Analysis Center, National Renewable Energy Laboratory, Golden, Colorado 80401, United States; ‡ Bio-Optimized Technologies to keep Thermoplastics out of Landfills and the Environment (BOTTLE) Consortium, Golden, Colorado 80401, United States; § Bioenergy Science and Technology Department, National Renewable Energy Laboratory, Golden, Colorado 80401, United States

**Keywords:** environmental justice, circular economy, sustainability

## Abstract

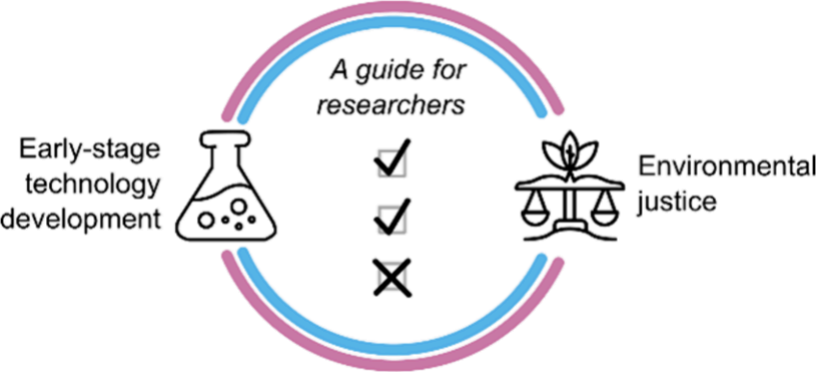

The circular economy could transform how industry and society approach resources and waste, resulting in significant environmental justice (EJ) implications. However, there are few resources for analyzing the EJ impacts of new circular economy technologies before they are deployed. This work presents an EJ framework tailored for early stage circular economy technologies and showcases its capabilities through a case study on enzymatic plastic recycling. By providing concise, actionable, and accessible guidelines based on technology readiness levels and a series of 20 questions, the framework empowers both experts and nonexperts to evaluate the justice implications of circular economy solutions. Preliminary user feedback highlights the approachability of the framework and its corresponding interactive worksheet, as well as their potential to stimulate innovative thinking toward a more just and sustainable future.

## Abstract 2

This work offers a practical framework and worksheet for identifying environmental justice impacts in early stage circular economy research.

## Introduction

Environmental justice (EJ) guarantees people’s agency over decisions that impact their fundamental human right to a clean, healthy, and sustainable life.^1^ EJ, as defined by the United States (U.S.) Environmental Protection Agency (EPA), encompasses the fair treatment and meaningful involvement of all people in the development, implementation, and enforcement of environmental laws, regulations, and policies.^2^ The circular economy, in which resources are kept in circulation rather than permitted to become waste, could have significant EJ implications.^3,4^ Globally, the waste sector offers low wages and poor working conditions, involves an estimated 15 million informal waste pickers, and releases, air, water, and solid emissions to predominantly disadvantaged communities.^5,6^ As circular economy technologies aim to displace the traditional waste sector, a consideration of EJ can help ensure that new innovations redress rather than perpetuate existing harms. When EJ is ignored, technologies could jeopardize the wellbeing and sustainability of communities, cause damage that must be mitigated or repaired, face social acceptance barriers, and miss opportunities that only become apparent through the inclusion of diverse perspectives.^3,7^

Evaluating EJ for emerging circular economy technologies can prove challenging. While several frameworks, such as EJScreen and social life cycle assessment (S-LCA),^8,9^ have been developed, they require geospatial information that is unlikely to be available at the earliest stages of technology development and calculate dozens to hundreds of indicators that may be difficult for nonexperts to interpret. The recently developed Justice Underpinning Science and Technology Research (JUST-R) framework strives to overcome similar challenges with regards to energy justice and early technology readiness level (TRL) renewable energy technologies.^10^ Users of JUST-R stated that the 40 recommended energy justice metrics helped to broaden their perspectives on their research, but also cited barriers to using the framework such as time requirements, insufficient resources to evaluate certain metrics, redundant or irrelevant metrics, and a lack of connection between the metrics and the broader energy justice concerns they were aiming to address.^11^

To guide circular economy development from the earliest stages of research and enable better mitigation of and planning for potential justice issues, EJ frameworks should be concise, actionable, and accessible to experts and nonexperts alike. Here, we present an inquiry-based framework for evaluating the EJ implications of circular economy technologies. The framework considers environmental, worker, supply chain, economic, and community impacts that can be evaluated qualitatively or quantitatively, depending on the TRL of the analyzed technology. For early stage technologies, we provide a simple worksheet comprising questions around key environmental, worker, and supply chain considerations. Through a case study on enzymatic plastic recycling, we show how the worksheet can identify justice issues and help to brainstorm solutions. This work provides researchers in the circular economy space with an accessible tool to incorporate preliminary EJ thinking into their technology development process and guide the transition to a more just and sustainable future.

## Results

### Framework Development

The EJ framework for circular economy technologies was developed based on the literature,^12,13^ existing frameworks and tools,^8,10^ and discussion with internal and external EJ experts. We first compiled a list of 26 EJ indicators related to (1) environmental impacts–health and safety impacts (qualitative), widely known social and sustainability issues (qualitative), existence of “greener” alternatives (qualitative), life cycle assessment (LCA) impacts including smog formation, respiratory effects, and human toxicity (quantitative), (2) worker impacts–use of child or forced labor (qualitative), occupational health and safety (quantitative), workers receiving unfair salaries (qualitative), workers with unfair hours (qualitative), respect of bargaining rights (qualitative), (3) supply chain impacts–existence of end-of-life management infrastructure (qualitative), (4) economic impacts–overall economic impacts (quantitative), affordability (quantitative), number and types of jobs created (quantitative), and (5) community impacts–privacy concerns (qualitative), transparency concerns (qualitative), history of problematic impacts or land use (qualitative), presence of an engagement plan (qualitative), land-use permits (qualitative), land-use conflicts (qualitative), land-use community preferences (qualitative), access to information (qualitative), respect of intellectual property rights (qualitative), social responsibility engagements (qualitative), community relation budget (quantitative), presence of disadvantaged communities (quantitative), access to material resources (quantitative).

**Figure 1:**
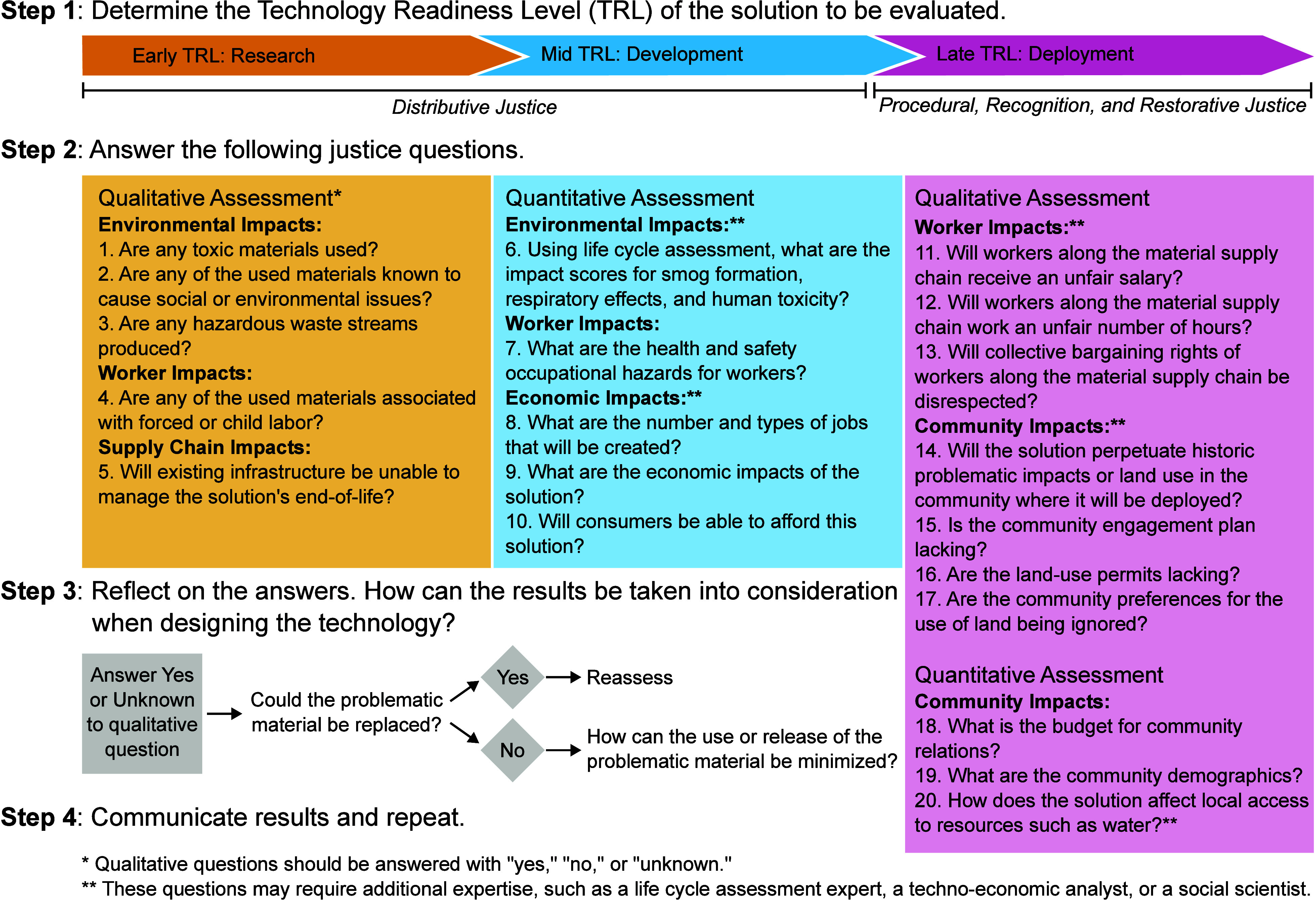
Framework for evaluating justice indicators at early, middle, and late technology readiness levels.

We then translated the indicators–combining where appropriate–into 20 questions and mapped these questions to different stages of the technology development process based on the technical, economic, environmental, and social information expected to be available at a given TRL (Figure 1). TRL estimates the maturity of a given technology on a scale of one to nine.^14^ Here, we define early TRL (1–3) as the research phase in which a scientific concept is first observed and experimentally validated; mid TRL (4–6) as the development phase in which the prototype technology is further tested in relevant environments; and late TRL (7–9) as the deployment phase in which the technology is demonstrated in an operational environment at or near full-scale. All questions for early TRL solutions require qualitative answers, as it is not anticipated that there will be sufficient quantitative data to conduct a full LCA and techno-economic analysis (TEA) or to know where the solution will be deployed. As the technology develops, more information is expected to become available and more quantitative indicators can be used. The early and mid TRL questions are primarily concerned with distributive justice, or the equitable distribution of the benefits and burdens of a technology, as potential risks can already be identified at this stage.^15^ The late TRL questions incorporate aspects of procedural justice, recognition justice, and restorative justice that are more relevant to facility siting, such as which stakeholders are heard and granted authority in the decision-making process of implementing a solution and how the solution can repair past harms in the community.^15,16^

Users of the framework should first identify the TRL of the solution to be evaluated and then proceed to answer the relevant questions using the resources provided in the “Case Study” section and the Supporting Information. Early TRL questions can be answered independently, but it is recommended to initiate collaborations with LCA and TEA practitioners, community engagement experts, and citizen advisory boards to enable robust EJ evaluation at mid and late TRLs.^17^ The boundaries between early, mid, and late TRL questions are meant to provide guidance but not to be rigid; if a user has the resources and expertise to address questions listed under a TRL higher than the solution being evaluated, they may do so. During mid- and late-stage technology development, users should also reassess the questions answered in the previous stage. Qualitative questions can be answered with a “yes,” “no,” or “unknown,” where “yes” or “unknown” prompt further investigation. Importantly, the framework encourages researchers to reflect on their answers and develop an action plan for addressing any justice issues that arise when working through the questions. Once action has been taken, the researchers should reevaluate their technology with the EJ framework to determine if the identified justice issues have been resolved.

### Case Study

**Figure 2:**
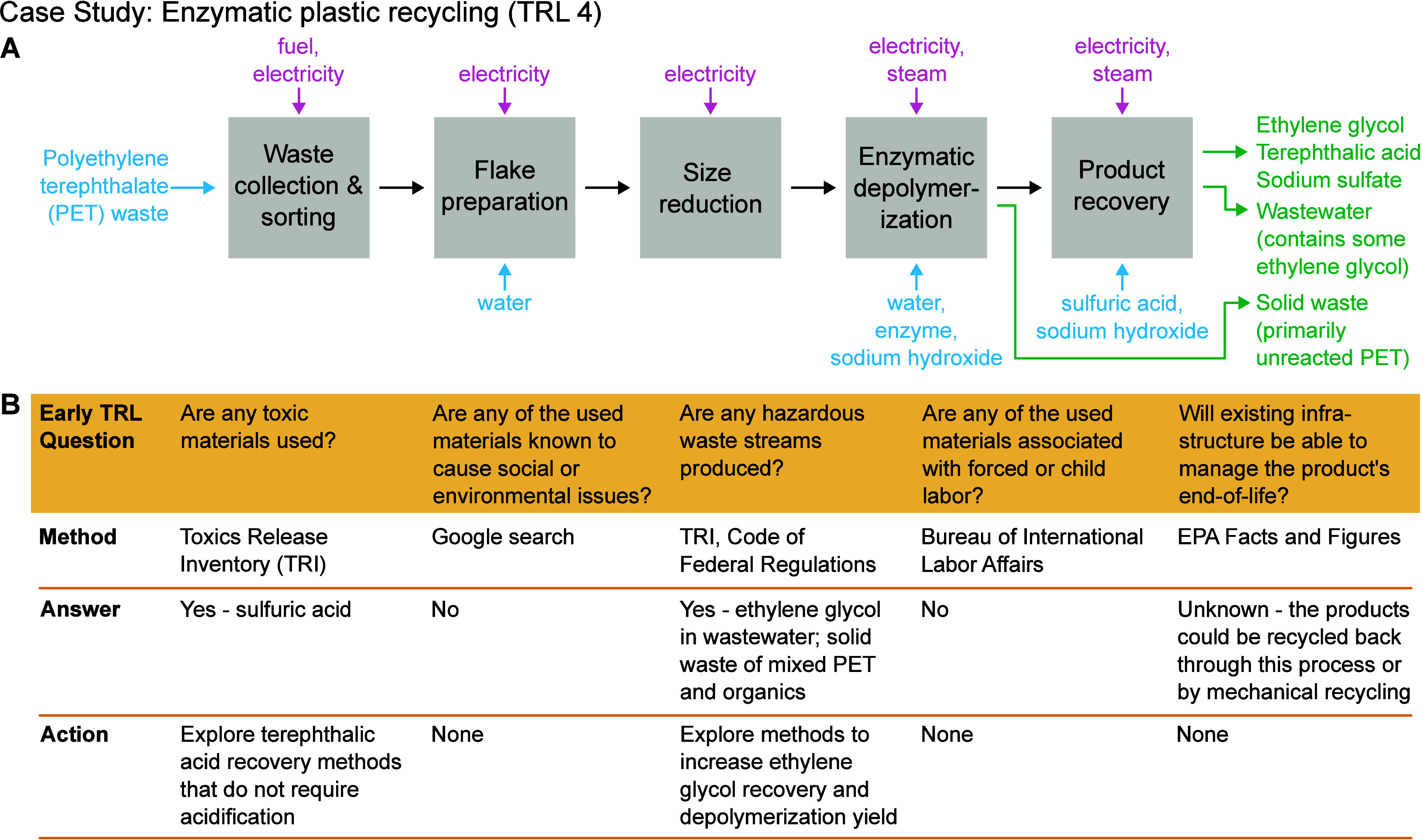
Case study applying the justice evaluation framework (early TRL section) to an enzymatic plastic recycling process. (A) Simple block flow diagram of enzymatic recycling, including material inputs (blue text), energy inputs (pink text), and material outputs (green text). (B) Results from the early TRL worksheet, including identified justice issues and potential innovations to overcome them.

To guide early TRL assessment using this EJ framework, we developed a simple worksheet (see Supporting Information) and applied it to a case study of enzymatic polyethylene terephthalate (PET) recycling (Figure 2). Enzymatic hydrolysis uses enzymes to depolymerize waste PET into its monomers, ethylene glycol and terephthalic acid, which can then be repolymerized into high quality PET products such as water bottles.^18^ As this circular economy solution has been demonstrated at the pilot scale but not widely deployed,^19^ we consider it to be at mid TRL.

The worksheet first directs users to prepare a block flow diagram of the process to be analyzed, including material and energy inputs and outputs, as demonstrated in Figure 2A for enzymatic recycling. Quantification of these flows is optional for the early TRL analysis stage. Users are then directed to five early TRL EJ questions (Figure 2B). Based on trials with researchers external to the project team, the worksheet is estimated to take approximately 1 h to complete.

The first environmental impact question asks: “Are any toxic materials used?” Understanding toxic chemical use in early stage processes is critical as the use of hazardous chemicals will directly affect the safety of workers at a future upscaled facility.^20^ If those chemicals are not managed properly and escape the facility, they will impact the health of local communities and environments.^21^ It is also important to plan for proper safety and regulation, which could increase the cost of a process at-scale. All input materials should be searched in the Chemical List provided by the U.S. EPA’s Toxics Release Inventory (TRI),^22^ which documents toxic chemical releases and pollution prevention activities.^23^ In certain cases, it may be necessary to use proxy chemicals for more specialized materials (e.g., lead rather than lead iodide). For enzymatic recycling, sulfuric acid was flagged in the TRI. Sulfuric acid is used to acidify the reaction solution after enzymatic hydrolysis to precipitate the monomer terephthalic acid for recovery. It would be challenging to replace sulfuric acid as all strong acids are listed in the TRI. However, the process could be reconfigured to eliminate the acidification step; it has been reported that using a high PET concentration during enzymatic depolymerization can enable terephthalic acid to precipitate without the addition of sulfuric acid.^24,25^ Once action has been taken on this justice question, the user is encouraged to reevaluate the process.

To answer the second environmental and social impact question “Are any of the used materials known to cause social or environmental issues?”, a Google search is recommended. Using a search structure of “material” + “social impacts” and “material” + “environmental health” for each input material identified in the block flow diagram, we screened the first ten sources for each search. This procedure, although simple, can help identify widely known ethical issues, such as worker exploitation in cobalt mining or the harmful health effects of per- and poly fluoroalkyl substances, thereby providing a “sanity check” of the eventual implications of the studied process. For enzymatic recycling, no widely known social or environmental issues were identified.

The third environmental impact question is “Are any hazardous waste streams produced?” When the technology of interest is upscaled, any hazardous waste generated by the process must be properly managed at the facility or at partner facilities; otherwise, it could be released into the environment and affect the health of local communities. Emissions to air and water should be searched in the TRI. For enzymatic recycling, ethylene glycol is emitted to wastewater, which is listed as toxic in the TRI if the concentration is greater than one volume percent.^22^ Although ethylene glycol cannot be eliminated because it is a coproduct of the recycling process, its emission could be minimized by increasing ethylene glycol recovery during downstream separations and by recycling the wastewater back through the depolymerization reactor. Organic and solid waste streams should be investigated in the Code of Federal Regulations.^26^ The solid waste stream generated by enzymatic recycling primarily comprises unreacted PET and other plastic contaminants and is not expected to be treated as hazardous waste. However, waste disposal to landfill still represents a social and economic burden and could be minimized by increasing the depolymerization yield of the recycling process.

“Are any of the used materials associated with forced or child labor?” is the only workers-related question asked at the early TRL stage. The International Labor Organization estimates that 160 million children were engaged in child labor worldwide in 2021, 50% of which were in hazardous labor.^27^ This question can help guide decisions early in the innovation process to minimize the use of materials associated with child or forced labor. All input chemicals and materials should be searched in the Bureau of International Labor Affair’s List of Goods Produced by Child Labor or Forced Labor.^28^ For enzymatic recycling, no input chemicals were identified as problematic.

Lastly, the question “Will existing infrastructure be able to manage the product’s end-of-life?” encourages researchers to consider the disposal strategy for the proposed solution. Landfills and incineration facilities are overwhelmingly located close to disadvantaged communities.^6^ Avoiding these waste management strategies and littering, as well as designing products for recycling from the early stages of innovation, can help reduce burdens on local communities. Information on the end-of-life management and littering of consumer products can be obtained through the U.S. EPA’s Web site^29^ and litter reports,^30^ while estimating the end-of-life of other more specialized products may require additional searches. Enzymatic recycling is an end-of-life technology. However, it does produce monomers that can be used to make PET products, approximately 76% of which were landfilled, 9% incinerated, and 15% recycled in the U.S. in 2019.^31^ A key target of this technology should therefore be to loop PET through enzymatic recycling multiple times in order to avoid its loss to landfills (currently it is estimated that PET could be recycled 4 times through enzymatic hydrolysis while retaining sufficiently high polymer quality).^32^

These early TRL questions identified sulfuric acid, ethylene glycol emissions, and waste generation as key justice concerns for enzymatic recycling. Neither sulfuric acid nor ethylene glycol recovery were flagged as problematic in previous LCA or TEA studies,^24,33^ highlighting the importance of a holistic evaluation approach that incorporates justice. Although the case study evaluated enzymatic recycling independently, a future comparison to conventional PET manufacturing and disposal could help identify existing EJ concerns that could be addressed by this circular economy solution.

Researchers interested in exploring the mid TRL questions for their technology are encouraged to build a more detailed process flow diagram and refer to further guidance on answering the mid TRL questions in the Supporting Information.

## Discussion

In July 2023, the early TRL EJ worksheet was piloted with 80 polymer chemists and chemical engineers from four American national laboratories and five American and British academic institutions that were members of the U.S. Department of Energy funded Bio-Optimized Technologies to keep Thermoplastics out of Landfills and the Environment (BOTTLE) Consortium. The participants comprised approximately 50% early career researchers (students, postdoctoral researchers, and interns) and 50% midcareer to senior researchers, of which 40% identified as female and 60% male and 5% self-identified as an underrepresented ethnic minority in science. Although this breakdown by sex and ethnicity is consistent with statistics reported for the U.S. science and engineering workforce by the National Science Foundation,^34^ the pilot group was not representative of the U.S. population at-large and therefore may not have captured all relevant viewpoints.

After a brief introduction to EJ concepts, researchers worked in groups of five to ten people to draw a simple block flow diagram of a technology that one or more of them were investigating and to answer at least two of the questions in the worksheet. After 30 min, the groups were asked to provide feedback to the moderators (the authors of this study). Overall, most groups reported that the worksheet was intuitive, included useful resources for answering the questions, and sparked further discussion and innovation that may not have occurred otherwise. For example, one team explored a chemical recycling process for nylon that does not use organic solvents but requires a tungsten catalyst. Originally, the researchers assumed that the process would be beneficial from an EJ perspective because of the lack of organic solvents. However, through the worksheet, the team discovered that tungsten ore mining is associated with child and forced labor in the Democratic Republic of the Congo,^28^ and they began to consider strategies for catalyst recycling or using more benign metals. Another team focused on a chemical recycling process for mixed textile waste containing cotton, PET, and nylon. The process was found to generate a hazardous waste stream containing dyes and additives such as per- and poly fluorinated substances (PFAS). These chemicals originated from the waste textile feedstock and therefore could not be avoided, so the researchers developed an action plan for converting the hazardous chemicals into benign derivatives and for considering suitable downstream engineering controls.

The framework and corresponding worksheet developed in this work can help stimulate EJ thinking without requiring extensive skill development or onboarding of external experts. However, they are not comprehensive. The framework simplifies justice issues into a set of questions, which cannot fully capture the complexity and nuances of real-world justice concerns in different geographic and socioeconomic contexts. In some cases, the multifaceted nature of EJ may be oversimplified. The framework also assumes that, even if quantitative data and geospatial information are unavailable, qualitative data will be accessible. The ability to obtain even qualitative information may vary across technologies and regions, affecting the usability of the framework. Lastly, we presented a single case study focused on enzymatic plastic recycling in the U.S., and the generalizability of the findings to other circular economy technologies, renewable technologies more broadly, or other geographies may be limited. Next steps should include validation of the framework with a more diverse set of researchers and other stakeholders for a broader suite of sustainable innovations, extension of the evaluation methodology for the mid and late TRL questions, integration with decision-making processes, and long-term monitoring of justice outcomes from technologies that leverage this framework.

## Conclusion

Incorporating EJ considerations into early stage circular economy research and development can ensure that scientific innovations truly provide benefits to everyone and do not negatively impact certain communities. This work presented an accessible, actionable, inquiry-based framework for investigating the EJ implications of emerging circular economy technologies at various stages of the research and development process. The framework included a six-part worksheet to help researchers qualitatively evaluate the environmental and worker impacts of their early TRL technology and brainstorm solutions to address any arising justice concerns. A case study on enzymatic plastic recycling showed how the EJ worksheet could identify problematic areas such as sulfuric acid use and ethylene glycol emissions that did not appear in other LCA and TEA analyses. While this EJ evaluation framework does not capture all aspects of the diverse justice space, the developed tools can help spark holistic thinking toward the development of a just circular economy.

## Data Availability

All data are available in the main text or the Supporting Information.

## Supporting Information Available

Early TRL worksheet used for the case study presented in this work. Readers are encouraged to try the worksheet for their own technologies (PDF)

## References

[ref1] United Nations General Assembly. Human Right to a Clean, Healthy and Sustainable Environment: Resolution/Adopted by the General Assembly; 2022. https://digitallibrary.un.org/record/3983329?ln=en&v=pdf (accessed 2024–04–29).

[ref2] Learn About Environmental Justice | US EPA. https://www.epa.gov/environmentaljustice/learn-about-environmental-justice (accessed 2024–01–18).

[ref3] Ashton W. S.; Fratini C. F.; Isenhour C.; Krueger R. Justice, Equity, and the Circular Economy: Introduction to the Special Double Issue. Local Environ. 2022, 27, 1173–1181. 10.1080/13549839.2022.2118247.

[ref4] Amorim de Oliveira C. D. Environmental Justice and Circular Economy: Analyzing Justice for Waste Pickers in Upcoming Circular Economy in Fortaleza, Brazil. Circ. Econ. Sustain. 2021, 1, 815–834. 10.1007/s43615-021-00045-w. 34888559 PMC8133516

[ref5] Kaza S.; Yao L.; Bhada-Tata P.; Van Woerden F. What a Waste 2.0 - A Global Snapshot of Solid Waste Management to 2050; Washington, D.C., 2018.

[ref6] Martuzzi M.; Mitis F.; Forastiere F. Inequalities, Inequities, Environmental Justice in Waste Management and Health. Eur. J. Public Health 2010, 20, 21–26. 10.1093/eurpub/ckp216. 20061348

[ref7] Zhang Z.; Lin Y. Impact of Perceived Social Justice on Public Acceptance toward Waste Disposal Facilities: Evidence from China. Environ. Impact Assess. Rev. 2023, 101, 107157 10.1016/j.eiar.2023.107157.

[ref8] EJScreen: Environmental Justice Screening and Mapping Tool | US EPA. https://www.epa.gov/ejscreen (accessed 2024–01–18).

[ref9] Engel-Cox J. A.; Wikoff H. M.; Reese S. B.; Jill Engel-Cox C. A. Techno-Economic, Environmental, and Social Measurement of Clean Energy Technology Supply Chains. J. Adv. Manuf. Process 2022, 4, e10131 10.1002/amp2.10131.

[ref10] Dutta N. S.; Gill E.; Arkhurst B. K.; Hallisey M.; Fu K.; Anderson K. JUST-R Metrics for Considering Energy Justice in Early-Stage Energy Research. Joule 2023, 7, 431–437. 10.1016/j.joule.2023.01.007.

[ref11] Arkhurst B. K.; Houghteling C. R.; Dutta N. S.; Clarke A.; Fu K.; Anderson K.; Gill E. Evaluating Energy Justice Metrics in Early-Stage Science and Technology Research Using the JUST-R Metrics Framework. Front Environ. Sci. 2023, 11, 1206013 10.3389/fenvs.2023.1206013.

[ref12] Traverso M.; Valdivia S.; Luthin A.; Roche L.; Arcese G.; Neugebauer S.; Petti L.; D’Eusanio M.; Tragnone B. M.; Mankaa R.; Hanafi J.; Benoit Norris C.; Zamagni A. Methodological Sheets for Subcategories in Social Life Cycle Assessment (S-LCA) 2021; United Nations Environment Programme (UNEP), 2021.

[ref13] SASB. https://sasb.org/ (accessed 2024–01–18).

[ref14] Office of Energy Efficiency and Renewable Energy. EERE 200.5: Technology Readiness Levels (TRLs); Washington, D.C., 2016. https://www.energy.gov/sites/prod/files/2016/07/f33/technology_readiness_levels.docx#:~:text=Technology%20Readiness%20Levels%20(TRLs)%3A,readiness%20level%20should%20be%20providedhttps://www.energy.gov/sites/prod/files/2016/07/f33/technology_readiness_levels.docx#:~:text=Technology%20Readiness%20Levels%20(TRLs)%3A,readiness%20level%20should%20be%20provided (accessed 2024–04–29).

[ref15] Dawson N.; Martin A.; Danielsen F. Assessing Equity in Protected Area Governance: Approaches to Promote Just and Effective Conservation. Conserv. Lett. 2018, 11, e12388 10.1111/conl.12388.

[ref16] Brandstedt E.; Brülde B. Towards a Theory of Pure Procedural Climate Justice. J. Appl. Philos. 2019, 36, 785–799. 10.1111/japp.12357.

[ref17] Ross L.; Day M. Community Energy Planning: Best Practices and Lessons Learned in NREL’s Work with Communities, 2022. https://www.nrel.gov/docs/fy22osti/82937.pdf (accessed 2024–04–29).

[ref18] Carniel A.; Waldow V. de A.; Castro A. M. de. A Comprehensive and Critical Review on Key Elements to Implement Enzymatic PET Depolymerization for Recycling Purposes. Biotechnol. Adv. 2021, 52, 107811 10.1016/j.biotechadv.2021.107811. 34333090

[ref19] Orlando M.; Molla G.; Castellani P.; Pirillo V.; Torretta V.; Ferronato N. Microbial Enzyme Biotechnology to Reach Plastic Waste Circularity: Current Status, Problems and Perspectives. Int. J. Mol. Sci. 2023, 24, 3877 10.3390/ijms24043877. 36835289 PMC9967032

[ref20] Schulte P. A.; McKernan L. T.; Heidel D. S.; Okun A. H.; Dotson G. S.; Lentz T. J.; Geraci C. L.; Heckel P. E.; Branche C. M. Occupational Safety and Health, Green Chemistry, and Sustainability: A Review of Areas of Convergence. Environ. Health 2013, 12, 1–9. 10.1186/1476-069X-12-31. 23587312 PMC3639149

[ref21] Johnston J.; Cushing L. Chemical Exposures, Health, and Environmental Justice in Communities Living on the Fenceline of Industry. Curr. Environ. Health Rep. 2020, 7, 48–57. 10.1007/s40572-020-00263-8. 31970715 PMC7035204

[ref22] Basic Search. https://guideme.epa.gov/ords/guideme_ext/f?p=guideme:chemical-list-basic-search (accessed 2024–01–18).

[ref23] Toxics Release Inventory (TRI) Program | US EPA. https://www.epa.gov/toxics-release-inventory-tri-program (accessed 2024–01–18).

[ref24] Uekert T.; DesVeaux J. S.; Singh A.; Nicholson S. R.; Lamers P.; Ghosh T.; McGeehan J. E.; Carpenter A. C.; Beckham G. T. Life Cycle Assessment of Enzymatic Poly(Ethylene Terephthalate) Recycling. Green Chem. 2022, 24, 6531–6543. 10.1039/D2GC02162E.

[ref25] Kaabel S.; Therien J. P. D.; Deschênes C. E.; Duncan D.; Friščić T.; Auclair K. Enzymatic Depolymerization of Highly Crystalline Polyethylene Terephthalate in Moist-Solid Reaction Mixtures. Proc. Nat. Acad. Sci. 2021, 118, e2026452118 10.1073/pnas.2026452118. 34257154 PMC8307448

[ref26] eCFR:: 40 CFR Part 261 Subpart D - Lists of Hazardous Wastes. https://www.ecfr.gov/current/title-40/chapter-I/subchapter-I/part-261/subpart-D (accessed 2024–01–18).

[ref27] International Labour Organization: UNICEF. Child Labour: Global Estimates 2020, Trends and the Road Forward; 2021.

[ref28] List of Goods Produced by Child Labor or Forced Labor | U.S. Department of Labor. https://www.dol.gov/agencies/ilab/reports/child-labor/list-of-goods-print (accessed 2024–01–18).

[ref29] National Overview: Facts and Figures on Materials, Wastes and Recycling | US EPA. https://www.epa.gov/facts-and-figures-about-materials-waste-and-recycling/national-overview-facts-and-figures-materials (accessed 2021–11–22).

[ref30] Burns-McDonnell; Cascadia; Salinas Davis LLC; Docking Institute. 2020 National Litter Study; 2021.

[ref31] Milbrandt A.; Coney K.; Badgett A.; Beckham G. T. Quantification and Evaluation of Plastic Waste in the United States. Resour. Conserv. Recycl. 2022, 183, 106363 10.1016/j.resconrec.2022.106363.

[ref32] Uekert T.; Singh A.; DesVeaux J. S.; Ghosh T.; Bhatt A.; Yadav G.; Afzal S.; Walzberg J.; Knauer K. M.; Nicholson S. R.; Beckham G. T.; Carpenter A. C. Technical, Economic, and Environmental Comparison of Closed-Loop Recycling Technologies for Common Plastics. ACS Sustain. Chem. Eng. 2023, 11, 965–978. 10.1021/acssuschemeng.2c05497.

[ref33] Singh A.; Rorrer N. A.; Nicholson S. R.; Erickson E.; DesVeaux J. S.; Avelino A. F. T.; Lamers P.; Bhatt A.; Zhang Y.; Avery G.; Tao L.; Pickford A. R.; Carpenter A. C.; McGeehan J. E.; Beckham G. T. Techno-Economic, Life-Cycle, and Socioeconomic Impact Analysis of Enzymatic Recycling of Poly(Ethylene Terephthalate). Joule 2021, 5, 2479–2503. 10.1016/j.joule.2021.06.015.

[ref34] National Center for Science and Engineering Statistics. Diversity and STEM: Women, Minorities, and Persons with Disabilities; Alexandria, 2023.

