# Supplementary material for: Strategies for Considering Environmental Justice in the Early-Stage Development of Circular Economy Technologies
Source: ACS Sustain Chem Eng. 2024 May 21;12(22):8307–12. doi: 10.1021/acssuschemeng.4c02205 (PMC11151278; doi:10.1021/acssuschemeng.4c02205)
Supplement: Supplementary file 1 — sc4c02205_si_001.pdf [file sc4c02205_si_001.pdf]

Supporting Information

Strategies for Considering Environmental Justice in the Early-Stage Development of Circular Economy Technologies

Taylor Uekert,[a,b,*] Julien Walzberg,[a] Hope M. Wikoff,[a] Meredith M. Doyle,[b,c] and Alberta C. Carpenter[a,b,*]

[a] Strategic Energy Analysis Center, National Renewable Energy Laboratory, Golden, CO 80401, United States

[b] Bio-Optimized Technologies to keep Thermoplastics out of Landfills and the Environment (BOTTLE) Consortium, Golden, CO 80401, United States

[c] Bioenergy Science and Technology Department, National Renewable Energy Laboratory, Golden, CO 80401, United States

* Corresponding authors: taylor.uekert@nrel.gov and alberta.carpenter@nrel.gov

This PDF contains: Worksheet for exploring the environmental justice impacts of early-stage technologies. For a continuously updated online version, please visit this link.

Environmental Justice Worksheet

Purpose: This worksheet aims to highlight aspects of your process(es), material(s), or technology(ies) that could raise environmental justice (EJ) concerns. It is specifically designed for early-stage research (i.e., technology readiness levels or TRLs of 1-3). EJ refers to a wide range of impacts on local communities, including those related to air pollution, access to clean water, jobs, wages, education, and more. While most EJ evaluation occurs at the deployment phase when a specific geographic location has been selected, it is also important to explore EJ during early-stage research to help guide the development of just and equitable technologies.

Have feedback or questions? We would love to hear from you! Please reach out to the team at taylor.uekert@nrel.gov

Step One: Prepare a block flow diagram of the process(es), material(s), or technology(ies) of interest in the space below. Include energy and material inputs and outputs, as well as any generated gas, liquid, or solid waste. Make sure to include all process steps necessary for your technology to function, such as any pretreatment or product purification steps. If available, you may include numerical values for these energy and material flows, but such detail is optional at this point. Now proceed to answer the following questions. *Note: See **Figure 2A** in the main text for an example block flow diagram.*

1. Does your process use toxic materials?

Procedure: Search for your input chemicals in the EPA's Toxics Release Inventory (TRI) database. You may need to use a simplified version of your material (e.g., lead rather than lead iodide).
https://guideme.epa.gov/ords/guideme_ext/f?p=guideme:chemical-list-basic-search

If your answer is No, continue to Question 2.

If your answer is Yes, list the chemical(s):

Could the above chemical(s) be replaced with non-TRI alternative(s)?

If your answer is Yes, list the alternative(s), conduct the same TRI search procedure, and include the results here. Need inspiration? Check out the EPA's Safer Chemical Ingredients List: https://www.epa.gov/saferchoice/safer-ingredients

If your answer is No, why must the chemical(s) be involved?

What are some management strategies for preventing the release of the chemical(s)?

Reflection Space: How will you take the above results into consideration when designing your process?

Why are we asking this question? *When your process is upscaled, the use of hazardous chemicals will directly affect the safety of workers at your facility. If these chemicals are not managed properly and escape the facility, they will impact the health of local communities and environments. It is also important to plan for proper safety and regulation as they will increase the cost of your process.*

2. Does your process use materials that have widely known social or environmental issues?

Procedure: Conduct a google search using "material" plus "social impacts" and "material" plus "environmental health." Look through the first 10 hits for each search. If any of the hits seem relevant, note below. Some phrases you might look for to indicate relevance include: exploitation, violence, habitat destruction, health risk, and human rights.

If your answer is No, continue to Question 3.

If your answer is Yes, list the material(s) and the corresponding social or environmental issues:

Could the above materials(s) be replaced with alternative(s)?

If your answer is Yes, list the alternative(s), conduct the same Google search procedure, and include the results here:

If your answer is No, how can you minimize the use of the material(s)?

Reflection Space: How will you take the above results into consideration when designing your process?

Why are we asking this question? *Searching for widely known social issues, like worker exploitation in cobalt mining, or environmental issues, like harmful health effects of per- and polyfluorinated substances (PFAS), is a good sanity check of the eventual implications of your process.*

3. Does your process generate any hazardous waste streams?

Procedure: Search (Ctrl + f) your solid and liquid waste streams in the Code of Federal Regulations (CFR). Search any chemicals that your process emits to air or to water in the EPA TRI database.

CFR: *https://www.ecfr.gov/current/title-40/chapter-I/subchapter-I/part-261/subpart-D*
TRI: *https://guideme.epa.gov/ords/guideme_ext/f?p=guideme:chemical-list-basic-search*

If your answer is No, continue to Question 4.

If your answer is Yes, list the type(s) of waste. Optionally, you can also provide an order of magnitude estimate of waste quantity (e.g., a higher quantity of waste than quantity of product, or a lower quantity of waste than quantity of product).

How could you minimize the quantity of waste being produced and/or prevent its release into the environment?

Reflection Space: How will you take the above results into consideration when designing your process?

Why are we asking this question? *When your process is upscaled, any hazardous waste must be properly managed at the facility, or it might be released into the environment and directly affect the health of local communities. Even if the waste is properly managed, over half of all communities located next to hazardous waste facilities are from disadvantaged backgrounds.*

4. Does your process use materials or substances that are widely known to be associated with child or forced labor?

Procedure: Search (Ctrl + f) your materials in the Bureau of International Labor Affairs database: *https://www.dol.gov/agencies/ilab/reports/child-labor/list-of-goods-print*

If your answer is No, continue to Question 5.

If your answer is Yes, list the material(s):

Could the above material(s) be replaced with alternative(s)?

If your answer is Yes, list the alternative(s), conduct the same search, and include the results here:

If your answer is No, how can you minimize the use of these materials?

Reflection Space: How will you take the above results into consideration when designing your process?

Why are we asking this question? *The International Labor Organization estimates that 160 million children were engaged in child labor worldwide in 2021, 50% of which were in hazardous labor. This question can help you make decisions early in the innovation process to minimize use of materials that are associated with child or forced labor.*

5. Once the product made by your process reaches its end-of-life, can it be managed by existing waste infrastructure?

Procedure: Consider whether your product might be landfilled, incinerated, or littered at its end-of-life. If your product is aimed at consumers, you can use the EPA's "Facts and Figures on Materials, Wastes and Recycling" to estimate how it will be managed at end-of-life. Note that plastic, cigarette butts, and paper are the most common littered items. For electronic waste, approximately 75% is currently sent to landfill and 25% to recycling.
https://www.epa.gov/facts-and-figures-about-materials-waste-and-recycling/national-overview-facts-and-figures-materials

If your answer is Yes, list the expected waste management strategy. If it is not recycling, are there ways you can redesign your product to enable recycling?

If your answer is No, are there ways to redesign your product to enable effective waste management?

Reflection Space: How will you take the above results into consideration when designing your process?

Why are we asking this question? *Landfills and incineration facilities are overwhelmingly located close to disadvantaged communities. Avoiding these waste management strategies and littering, as well as designing products for recycling from the early stages of innovation, can help reduce burdens on local communities.*

Recap. Look back through your Reflection Space answers throughout this worksheet. What are some of the main social and environmental pitfalls you should keep in mind as your technology progresses?

Feedback. Please use this space to write down any questions, concerns, confusion, or comments that arose while completing this worksheet.

Additional questions to consider at mid TRL:

Build a more detailed model of your process, including quantitative values for all material, energy, and waste flows. Material and waste flows can typically be measured as part of the experimental process. Energy flows can be estimated by checking equipment labels or user manuals for power requirements, using rule-of-thumb measures such as 1 kWh/m^3 for stirring, centrifugation, and filtration, or calculating $Q = mc\Delta T$ for thermal heating requirements (where m = mass, c = specific heat, and ΔT = change in temperature). Alternatively, process modeling software such as Aspen Plus or BioSTEAM can be used. Based on the quantified material and energy flows, conduct technoeconomic analysis (TEA) and life cycle assessment (LCA), ideally in collaboration with expert analysts to help streamline the process and ensure its accuracy. Use the results to revise your answers to questions 1-5 in the early TRL worksheet as well as to answer the following mid TRL questions.

6. What are the human health impacts of your process?

Procedure: Quantify toxicity, smog formation, and respiratory effects using an LCA method such as ReCiPe or TRACI, identify which process components are problematic, and propose strategies to replace or manage those components.

7. What are the health and safety occupational hazards for workers?

Procedure: Using incidence rates for the most closely related industry from the Department of Labor, estimate the incidence rates of workers for your process. Does this fall above or below the average? Consider whether you can change the process design to make it safer for workers.

8. What are the number and type of jobs that will be created?

Procedure: As part of the TEA, estimate the number and types of jobs that will be necessary at an upscaled facility for your process. Compare the number of jobs required for your process to those required by the conventional process it may be replacing.

9. What are the economic impacts of the solution?

Procedure: Using TEA estimates of jobs, estimate salaries from the Bureau of Labor Statistics (e.g., Chemicals, Average Hourly Earnings of Production Workers, input Series ID CEU323250008). Check that these salaries are above the national living wage and compare to average working person income.

10. Will consumers be able to afford this solution?

Procedure: Using TEA results, compare the cost of your process to the conventional process. If your process is comparable, it will likely be affordable for the same set of customers.

11. (Optional) Does this technology have the potential to violate consumer privacy?

Procedure: If the technology collects or holds data, the answer is yes. If not, the answer is no.
